# An “outer subarachnoid space”: fact or artifact? A commentary on “Structural characterization of SLYM– a 4th meningeal membrane” fluids and barriers of the CNS (2023) 20:93 by V. Plá et al.

**DOI:** 10.1186/s12987-024-00539-3

**Published:** 2024-06-03

**Authors:** Winfried Neuhuber

**Affiliations:** Institute of Anatomy, Friedrich-Alexander-University Erlangen-Nürnberg, Krankenhausstrasse 9, 91054 Erlangen, Germany

The classical two-layered structure of the leptomeninges, the arachnoid bordering the dura and the pia attached to the CNS surface was modified and extended over the past decades. The Liliequist membranes incompletely compartmentalize the basal cisterns in human [1], the arachnoid barrier cells (ABC) control transport between the dural border cells and the reticular arachnoid (e.g., [2]) and the intermediate lamella as defined by Krisch et al. [3] confines a perivascular compartment separated from the subarachnoid space. The description of the subarachnoid lymphatic-like membrane (SLYM) as a 4th meningeal membrane (numbered with respect to the triad “dura-arachnoid-pia”) subdividing the subarachnoid space into outer (oSAS) and inner (iSAS) compartments [4] may significantly remodel our anatomical, functional and pathophysiological ideas on the brain, its meninges and CSF dynamics. This study attempts to corroborate previous findings published by the same group [5]. Scrutinizing the present paper, I however stumbled over several oddities which seriously dampened my prima vista enthusiasm.

Studying the anatomy of delicate leptomenigeal membranes is notoriously difficult, and the authors are well aware of these problems. They point to the effect of hyperosmotic fixatives resulting in brain shrinkage and exaggerated wide leptomeningeal spaces. Delicate membranes might be displaced, e.g., the SLYM might be detached from the ABC thus covering the pia (their Fig. 1D). This apparently occurs to different extent at different places around the brain as can be seen in Figs. 3 and 6 taken from paraformaldehyde fixed and decalcified specimens. Thus, the SLYM seems “floating” between attaching to the ABC on one hand and to the pia on the other, with intermediate states in between. If the SLYM is proposed to divide the SAS into an outer and inner compartment, these should be highly variable in size and probably dynamically changing.

The caveat towards hyperosmotic fixatives applies even more to the EDTA solution mandatory for decalcification. A 10% EDTA solution as used by Plá et al. has an osmolarity of 806 mOsm (own measurements); i.e., almost three times the iso-osmolarity of about 300 mOsm. Particularly if applied over three weeks, this may significantly add to the displacement of leptomeningeal membranes.

The attachment of the SLYM to the ABC layer from the foramen magnum down is highly interesting. It was not investigated if this attachment extends throughout the spine. Nevertheless, it appears that meningeal anatomy may significantly differ between the cranial and spinal realms, the SAS being not subdivided in the latter. Tracer injections into the cisterna magna, penetrating below the foramen magnum the atlanto-occipital membrane with the attached dura, dural border cells, ABC layer and SLYM, necessarily reach the iSAS filling the basal cisterns (Fig. 2). Remarkably, no attempt was made in the present study to fill the oSAS with tracer as was done in the previous work [5]. From the considerations above, it is tempting to surmise that in the cranial cavity the SLYM was detached from its “normal” position on the inner surface of the ABC during extended hyperosmotic processing. Thus, the creation of separated oSAS and iSAS may be the result of unintended artifacts induced by potentiated hyperosmotic stress during both fixation and decalcification. Why the SLYM is not detached from ABC below the foramen magnum is not easily explained. One possible reason could be found in different surface areas of attachment. These are large in the cranium providing better opportunity for osmotic forces to work.

Although the authors laudably attempted to integrate their SLYM into the historical context, homologizing it (by coding green) to a pial layer in the Nabeshima / Orlin diagram (Fig. 8) (which rather resembles Krisch et al.’s intermediate lamella) is probably not justified.

Remarkably, at the end of the Discussion, the inner or reticular arachnoid layer was considered a possible synonym of SLYM. This may point to a solution of the enigma of a “4th meningeal membrane”: it probably represents nothing else other than the hyperosmotically detached reticular/inner arachnoid.

## Data Availability

Not applicable.

## Abbreviations

ABC: arachnoid barrier cells
CNS: central nervous system
CSF: cerebrospinal fluid
EDTA: ethylenediaminetetraacetic acid
iSAS, oSAS: inner and outer subarachnoid space, respectively
mOsm: milliosmol
SLYM: subarachnoid lymphatic-like membrane

## References

[1] Volovici V, Varvari I, Dirven CMF, Dammers R (2020). The membrane of liliequist– a safe haven in the middle of the brain. A narrative review. Acta Neurochir.

[2] Orlin JR, Osen KK, Hovig T (1991). Subdural compartment in pig: a morphologic study with blood and horseradish peroxidase infused subdurally. Anat Rec.

[3] Krisch B, Leonhardt H, Oksche A (1984). Compartments and perivascular arrangement of the meninges covering the cerebral cortex of the rat. Cell Tissue Res.

[4] Plá V, Bitsika S, Giannetto MJ, Ladron-de-Guevara A, Gahn-Martinez D, Mori Y, Nedergaard M, Mollgard K (2023). Structural characterization of SLYM– a 4th meningeal membrane. Fluids Barriers CNS.

[5] Mollgard K, Beinlich FRM, Kusk P, Miyakoshi LM, Delle C, Plá V, Hauglund NL, Esmail T, Rasmussen MK, Gomolka RS (2023). A mesothelium divides the subarachnoid space into functional compartments. Science.

